# Clinical characteristics and predictors of mortality in young adults with severe COVID-19: a retrospective observational study

**DOI:** 10.1186/s12941-020-00412-9

**Published:** 2021-01-06

**Authors:** Yanjiao Lu, Zhenli Huang, Meijia Wang, Kun Tang, Shanshan Wang, Pengfei Gao, Jungang Xie, Tao Wang, Jianping Zhao

**Affiliations:** 1 Department of Respiratory and Critical Care Medicine, National Clinical Research Center of Respiratory Disease, Tongji Hospital, Tongji Medical College, Huazhong University of Science and Technology, Wuhan, 430030 China; 2 Department of Respiratory and Critical Care Medicine, the First Affiliated Hospital of Henan University of Science and Technology, Luoyang, 471003 China

**Keywords:** COVID-19, Predictors, SARS-CoV-2, Severe, Young adults

## Abstract

**Background and objective:**

Little is yet known whether pathogenesis of COVID-19 is different between young and elder patients. Our study aimed to investigate the clinical characteristics and provide predictors of mortality for young adults with severe COVID-19.

**Methods:**

A total of 77 young adults with confirmed severe COVID-19 were recruited retrospectively at Tongji Hospital. Clinical characteristics, laboratory findings, treatment and outcomes were obtained from electronic medical records. The prognostic effects of variables were analyzed using logistic regression model.

**Results:**

In this retrospective cohort, non-survivors showed higher incidence of dyspnea and co-existing laboratory abnormalities, compared with young survivals in severe COVID-19. Multivariate logistic regression analysis showed that lymphopenia, elevated level of d-dimer, hypersensitive cardiac troponin I (hs-CTnI) and high sensitivity C-reactive protein (hs-CRP) were independent predictors of mortality in young adults with severe COVID-19. Further analysis showed that severely young adults with two or more factors abnormalities above would be more prone to death. The similar predictive effect of above four factors had been observed in all-age patients with severe COVID-19.

**Conclusion:**

Lymphopenia, elevated level of d-dimer, hs-CTnI and hs-CRP predicted clinical outcomes of young adults with severe COVID-19.

## Background

The newly emergent human severe acute respiratory syndrome coronavirus 2 (SARS-CoV-2) causes coronavirus disease 2019 (COVID-19), resulting in epidemics and pandemics [1, 2]. As of April 18th 2020, SARS-CoV-2 has caused more than 2,000,000 infections and 100,000 deaths worldwide [3].

Previous studies have focused on general epidemiological findings, clinical presentations, and clinical outcomes of patients of COVID-19 [4, 5]. Accumulating studies have suggested that all ages people are susceptible to SARS-CoV-2 infection, which can result in severe and even fatal respiratory diseases [6–8]. As reported by Liu et al., clinical features of elderly patients with COVID-19 were significantly different from that of younger patients [6]. It has to be noted that elderly patients were with more comorbidities, leading to more complicated pathogenesis in COVID-19 [8]. Would the pathogenesis of COVID-19 be different in young adults, with less comorbidities and more strong host immune? Indeed, there are plenty of severe COVID-19 cases in young adults. However, the characteristics and associated risk factors of non-elderly patients, especially with severe COVID-19, have not been fully elucidated so far. Furthermore, young people as the main social labor force, it is urgent to identify the risk factors associated with mortality of young adults in severe COVID-19.

We intended to investigate the clinical characteristics and provide predictors of mortality for young adults with severe COVID-19.

## Methods

### Study participants and data collection

For this retrospective, non-interventional study, a total of 376 patients with COVID-19 were recruited retrospectively at Tongji Hospital from January 25 to February 15, 2020, and 299 cases were excluded due to ineligible. According to final outcome, we classified 77 young adults with severe COVID-19 into survivor group (37patients) and non-survivor group (40 patients).

The study was performed in accordance with Tongji Hospital Ethics Committee (TJ-IRB20200353). Written informed consent was waived by the Ethics Commission owing to the rapid emergence of this infectious disease.

We prospectively collected information of all patients including demographic data, clinical characteristics, laboratory findings, treatment and outcomes from reviewing medical records. Three researchers individually checked the collected data. To investigate the risk of in-hospital death, all patients were followed from admission to discharge or death (1–58 days). The primary outcome was death during hospitalization.

### Definitions

The diagnosis of COVID-19 was created according to the definition established by World Health Organization (WHO) interim guidance [9]. The clinical classifications of patients as having severe or not COVID-19 are established based on the *2019 American Thoracic Society / Infectious Disease Society of America guideline*, taking into account its global acceptance for severity stratification of community-acquired pneumonia although lacking of validation in patients with viral pneumonia [10]. The young adults were defined as people under age of 65 years old.

### Primary variables selection in logistic regression model

Univariate and multivariate logistic regression were performed to make out the association of clinical characteristics and laboratory parameters for the risk of death. Taking the total deaths events (n = 40) of our study into account and to avoid overfitting in multivariate logistic regression model, four factors were chosen for multivariable logistic analysis on the basis of previous results and clinical constraints. Original researches have shown plasma levels of d-dimer and high sensitivity cardiac troponin I (hs-CTnl) to be higher in severe or critical ill cases, whereas lymphopenia has been less observed in surviving or moderate ill patients [11–13]. Therefore, we chose lymphocyte count, d-dimer, hs-CTnl, and other variable as the four variables in our multivariable logistic regression model. According to the AIC level of each model, we chose the best regression model.

We ruled out variables from the multivariable analysis if the differences between-group were not significant, if they had collinearity, if the accuracy was not confirmed (eg, exposure, which was self-reported), and if the number of incidences was too small to calculate odds ratios [11].

According to the level of lymphocyte, d-dimer, hs-CTnI and high sensitivity C-reactive protein (hs-CRP), we classified of young adults with severe COVID-19 to subgroups. For each factor, cut points used to define a high level were as following: Lymphocyte < 0.5 × 10^9^/L, d-dimer > 21 μg/mL, hs-CTnI > 15.6 pg/ml and hs-CRP > 100 mg/L. High-risk group indicated elevation in two or more factors, while low-risk group indicated elevation in one or no factors.

### Statistical analysis

We described the categorical variables as frequency rates and percentages, and continuous variables median and interquartile range (IQR) values. Unpaired 2-sided Student’s t test was used for continuous variables if the data were normally distributed; if not, Mann–Whitney test was used. The frequencies of categorical variables were compared using χ2 test or Fisher’s exact test as appropriate.

All statistical analyses and graphs were generated and plotted using SPSS (version 22.0) and GraphPad Prism version 7.0 software (GraphPad Software Inc). P < 0.05 was considered statistically significant.

## Results

### Demographics and baseline characteristic of young adults with severe COVID-19

From 25 Jan 2020 to 15 Feb 2020, 376 patients were admitted to Tongji hospital with confirmed COVID-19, of whom 299 were considered ineligible. 77 young adults with severe COVID-19 were included in this study (Additional file 1: Figure S1). Baseline characteristics of patients were divided into subgroups by survival or non-survival (Table 1). Different from all-age populations, there were no significant difference in age and sex among young adults with severe COVID-19 (Additional file 1: Table S1). Patients in non-survivor group were with faster heart rate than survival group. Other characteristics such as exposure history, smoking history, comorbidities, respiratory rate, percutaneous oxygen saturation, blood pressure showed no significance between two groups.

**Table 1:** Demographics and baseline characteristic of young adults with severe COVID-19

	Total n = 77	Survivor n = 37	Non-survivor n = 40	P value
Age, years	59 (54–63)	58 (50–62)	60 (57–64)	0.077
Sex (male)	50 (65%)	21 (57%)	29 (73%)	0.148*
Exposure history	10 (13%)	5 (14%)	5 (13%)	0.895*
Smoker	3 (4%)	2 (6%)	1 (3%)	0.981*
Comorbidity	72 (94%)	35 (95%)	97 (93%)	1*
Hypertension	26 (34%)	12 (33%)	414 (35%)	0.812*
Diabetes	9 (12%)	5 (14%)	4 (10%)	0.901*
Coronary heart disease	3 (4%)	0 (0)	3 (8%)	0.241*
Malignancy	2 (3%)	2 (6%)	0 (0)	0.228*
Chronic kidney disease	2 (3%)	0 (0)	2 (5%)	0.494*
Tuberculosis	1 (1%)	0 (0)	1 (3%)	1*
Chronic hepatitis B	4 (5%)	2 (5%)	2 (5%)	1*
Others	28 (36%)	14 (38%)	14 (35%)	0.796*
Respiratory rate, > 30 breath per min	13/75 (17%)	4/37 (11%)	9/40 (24%)	0.141*
Heart rate, ≥ 125 beats per min	7/76 (9%)	0/36 (0)	7/40 (18%)	0.012*
Percutaneous oxygen saturation, ≤ 93%	55 (71%)	25 (68%)	30 (75%)	0.471
Systolic pressure, mmH	131 (119–146)	130 (114–142)	132 (121–151)	0.582
Diastolic pressure, mmHg	79 (67–85)	76 (62–85)	80 (71–88)	0.185
Fever	72 (94%)	35 (95%)	37 (93%)	1*
Sore throat	4 (5%)	2 (5%)	2 (5%)	1*
Cough	59 (77%)	27 (73%)	32 (80%)	0.467*
Chest pain	3 (4%)	1 (3%)	2 (5%)	1*
Dyspnea	51 (66%)	18 (49%)	33 (83%)	0.002*
Fatigue	42 (55%)	20 (54%)	22 (55%)	0.834*
Myalgia	14 (18%)	8 (22%)	6 (15%)	0.452*
Nausea or vomiting	5 (7%)	1 (3%)	4 (10%)	0.403*
Diarrhea	25 (33%)	12 (33%)	13 (33%)	0.995*
Stomachache	5 (5%)	1 (3%)	4 (10%)	0.403*
Headache	7 (9%)	3 (8%)	5 (10%)	1*
Unconscious	2 (3%)	0 (0)	2 (5%)	0.494*
Dizziness	3 (4%)	0 (0)	3 (8%)	0.241*

Data are median (IQR), n (%), or n/N (%). p values were calculated by Mann–Whitney U test, χ^2^ test, or Fisher’s exact test, as appropriate

*χ^2^ test comparing all subcategories

Similar to the results reported in previous researches, we pointed out that the top four symptoms included fever (94%), cough (77%), dyspnea (66%), fatigue (55%) in hospital among all-age population (Table 1, Additional file 1: Table S2) [1, 12]. Except for dyspnea that were more often present in non-survivor group than survivor group (83 vs. 49%), other symptoms were comparable in two groups. But in all-age patients, incidence of unconscious and dizziness were higher in non-survivors than that of survivors.

### Laboratory findings

The non-survivors had more white blood cells and neutrophils counts than that of the survivors, may result from the presence of secondary bacterial infection as indicated by higher concentrations of hs-CRP and procalcitonin (Table 2, Additional file 1: Table S3). As expected, the non-survivors had reduced lymphocytes. Compared with survivors, those in non-survivor group underwent susceptible to abnormalities of liver, kidney and coagulation function, suggested by elevation of albumin or creatinine, and dysregulation of d-dimer. The non-survivors had experienced more frequently and severe heart injury, as all laboratory heart function parameters including hs-CTnl, myoglobin, and N-terminal pro-brain natriuretic peptide (NT-proBNP), were all significantly increased. The similar results had been shown in all-age patients.

**Table 2:** Laboratory examinations of young adults with severe COVID-19

Findings (normal range)	Total n = 77	Survivor n = 37	Non-survivor n = 40	P value
Blood routine test				
White blood cell, × 10^9^/L (3.5–9.5)	7.5 (5.9–10.6)	6.5 (4.7–8.4)	9.7 (7.1–13.0)	< *0.001*
Neutrophil granulocyte, × 10^9^/L (1.8–6.3)	6.6 (4.2–9.7)	5.0 (3.6–6.7)	8.8 (5.9–12.0)	< *0.001*
Lymphocyte, × 10^9^/L (1.1–3.2)	0.7 (0.5–1.0)	0.9 (0.7–1.2)	0.6 (0.4–0.7)	< *0.001*
Red blood cell, × 10^9^/L (3.8–5.1)	4.2 (3.7–4.6)	4.2 (3.7–4.4)	4.2 (3.7–4.8)	0.383
Haemoglobin, g/L (130–175)	129 (115–140)	129 (118–136)	130 (111–143)	0.665
Platelet, × 10^9^/L (125–350)	194 (148–128)	201 (162–279)	153 (127–244)	0.092
Coagulation function				
PT, s (11.5–14.5)	14.9 (13.8–16.3)	13.8 (13.3–14.6)	15.7 (15.1–17.2)	< *0.001*
APTT, s (29.0–42.0)	39.9 (36.2–44.9)	40.5 (37.1–44.8)	39.4 (34.4–45.0)	0.468
D-dimer, μg/ml (< 0.5)	2.4 (1.0–21.0)	1.3 (0.7–2.2)	18.2 (3.0–21.0)	< *0.001*
Biochemical test				
Albumin, g/L (35.0–52.0)	31.1 (28.5–35.0)	32.8 (20.2–36.3)	30.0 (27.2–33.7)	*0.001*
Globulin, g/L (20.0–35.0)	34.6 (31.5–37.9)	33.6 (31.1–36.8)	35.5 (31.6–39.2)	0.172
Aspartate aminotransferase, U/L (≤ 40)	34 (23–52)	30 (20–46)	37 (28–57)	0.296
Alanine aminotransferase, U/L (≤ 41)	29 (20–50)	28 (20–57)	29 (18–48)	0.721
Total-bilirubin, umol/L (≤ 26)	10.1 (7.5–14.9)	8.6 (6.7–11.7)	12.2 (8.4–19.2)	*0.002*
Direct-bilirubin, umol/L (≤ 8)	4.9 (3.5–7.4)	3.6 (3.3–5.1)	6.4 (4.5–10.2)	< *0.001*
Creatinine, umol/L (59–104)	70 (55–87)	64 (50–82)	78 (59–99)	*0.024*
Urea nitrogen, mmol/L (3.1–8.0)	5.3 (3.5–7.2)	3.7 (2.9–5.2)	7.0 (5.3–9.4)	< *0.001*
LDH, U/L (135–225)	460 (347–585)	359 (268–456)	567 (475–663)	0.442
Infection-related biomarkers				
Procalcitonin, ng/mL (0.02–0.05)	0.13 (0.04–0.29)	0.04 (0.02–0.13)	0.23 (0.14–0.60)	< *0.001*
ERS, mm/h (0–15)	35 (20–64)	34 (20–71)	37 (20–54)	0.586
Ferritin, ng (30–400)	1336 (685–2020)	1113 (374–1605)	1701 (995–3113)	0.032
hs-CRP, mg/L (< 1)	72.5 (38.3–140.2)	52.1 (28.4–88.4)	120 (55.9–183.0)	< *0.001*
Myocardial enzymes				
Creatine kinase, U/L (≤ 190)	116 (54–308)	100 (42–212)	119 (61–397)	0.442
NT-BNP, pg/mL (< 285)	292 (87–852)	87 (39–205)	709 (300–1773)	< *0.001*
hs-CTnl, pg/mL (≤ 15.6)	13.0 (3.4–111.3)	3.6 (2.1–10.1)	41.5 (12.1–308.6)	< *0.001*
Myoglobin, ng/mL (≤ 106)	131 (29–324)	29 (21–123)	258 (130–470)	*0.001*

*PT* prothrombin time, *APTT* activated partial thromboplastin time, *LDH* lactate dehydrogenase, *ERS* erythrocyte sedimentation rate, *hs-CRP* high sensitivity C-reactive protein, *NT-proBNP* N-terminal pro-brain natriuretic peptide, *hs-CtnI* hypersensitive cardiac troponin I. Data are median (IQR), n (%), or n/N (%). p values were calculated by Mann–Whitney U test, χ^2^ test, or Fisher’s exact test, as appropriate

*χ^2^ test comparing all subcategories

### Treatment and outcomes

More than half non-survivors experienced mechanical ventilation and ICU admission (Table 3, Additional file 1: Table S4). The median time from illness onset to death was 24 days (IQR 6–17), whereas the median time from illness onset to discharge was 38 days (IQR 21–33). The similar trend was shown in hospital length of stay (11 [IQR 6–17] vs. 25 [IQR 21–33]). Consistent with discharge standers, viral shedding of survivors was happened during treatment course.

**Table 3:** Treatment and outcomes of young adults with severe COVID-19

	Total n = 77	Survivor n = 37	Non-survivor n = 40	P value
*Treatment*				
High-flow nasal cannula oxygen therapy	15 (20%)	6 (16%)	9 (23%)	0.487*
Non-invasive mechanical ventilation	28 (36%)	2 (5%)	26 (65%)	< 0.001*
Invasive mechanical ventilation	27 (35%)	0 (0)	27 (68%)	< 0.001*
ECMO	2 (3%)	0 (0)	2 (5%)	0.494*
*Outcomes*				
ICU admission	29 (38%)	1 (3%)	28 (70%)	< 0.001*
ICU length of stay, days	10 (4–19)	.	10 (4–17)	.
Hospital length of stay, days	19 (11–27)	25 (21–33)	11 (6–17)	< *0.001*
Time from illness onset to ICU admission, days	16 (13–20)	.	16 (13–21)	.
Time from illness onset to death or discharge, days	30 (22–39)	38 (32–45)	24 (19–29)	< *0.001*
SARS-CoV-2 RT-PCR test (-)	43/77 (56%)	35/37 (95%)	8/40 (20%)	< 0.001*
Duration of viral shedding after COVID-19 onset, days	22 (18–27)	23 nnnn	19 n	0.054

Data are median (IQR), n (%), or n/N (%). P values were calculated by χ^2^ test, or Fisher’s exact test, as appropriate

*ECMO* extracorporeal membrane oxygenation, *ICU* intensive care unit, *RT-PCR* real-time polymerase chain reaction

*χ^2^ test comparing all subcategories

### Predictors of mortality

All demographic data, clinical symptoms, laboratory findings, treatments and outcomes were shown in Tables 1 and 2, we evaluated every variable that demonstrated statistical significance with p < 0.05 between non-survivor and survivor groups by univariate logistic regression analysis (Table 4). Slightly different from all-age population, white blood cell, neutrophil granulocyte, lymphocyte, prothrombin time, d-dimer, albumin, direct-bilirubin, urea nitrogen, procalcitonin, hs-CRP, NT-proBNP and hs-CTnl were associated with the risk of mortality (Additional file 1: Table S5).

**Table 4:** Univariate logistic regression analysis of mortality risk factors in young adults with severe COVID-19

Factor	Univariable OR (95% CI)	P value
White blood cell > 9.5 × 10^9^/L, %	5.167 (1.716–15.087)	*0.003*
Neutrophil granulocyte > 6.3 × 10^9^/L, %	7.118 (2.608–19.428)	< *0.001*
Lymphocyte < 0.5 × 10^9^/L, %	9.273 (2.441–35.227)	*0.001*
PT > 14.5 s, %	21.778 (6.554–72.367)	< *0.001*
D-dimer > 21 μg/mL, %	34.200 (4.256–274.808)	*0.001*
Albumin < 30 g/L, %	7.143 (2.519–20.257)	< *0.001*
Direct-bilirubin > 8umol/L	5.457 (1.410–21.117)	*0.014*
Urea nitrogen > 8 mmol/L	4.857 (1.246–18.933)	*0.023*
Procalcitonin > 0.05 ng/mL, %	19.559 (4.077–93.825)	< *0.001*
hs-CRP > 100 mg/L, %	5.955 (2.098–16.904)	*0.001*
NT-proBNP ≥ 285 pg/ml, %	18.000 (5.211–62.176)	< *0.001*
hs-CTnI > 15.6 pg/ml, %	16.667 (4.234–65.601)	< *0.001*

*OR* odds ratio, *PT* prothrombin time, *hs-CRP* high sensitivity C-reactive protein, *NT-proBNP* N-terminal pro-brain natriuretic peptide, *hs-CtnI* hypersensitive cardiac troponin I

Multivariate logistic regression analyses were applied to assess the independent prognostic effect of related factors (Table 5). Before adjusting other variables, odds ratio (OR) of lymphocyte < 0.5 × 10^9^/L, d-dimer > 21 μg/mL and hs-CTnI > 15.6 pg/ml were 7.03 (95% CI 1.435–40.275), 11.012 (95% CI 1.092–111.100) and 13.876 (95% CI 2.888–66.673). When adjusting for other variables, the OR of above three fixed variables changed slightly. According to the AIC level of each model (Additional file 1: Table S6), we chose the lowest AIC level as the best. The multivariate logistic regression analyses model 5 including lymphocyte < 0.5 × 10^9^/L, d-dimer > 21 μg/mL, hs-CTnI > 15.6 pg/ml and hs-CRP > 100 mg/L (Table 5, mode 5), were the best predictors of mortality.

**Table 5:** Multivariate logistic regression analysis of mortality risk factors in young adults with severe COVID-19

Mode	Multivariable OR (95% CI)	P value
*Mode 1*		
Lymphocyte < 0.5 × 10^9^/L, %	7.03 (1.435–40.275)	*0.017*
D-dimer > 21 μg/mL, %	11.012 (1.092–111.100)	*0.042*
hs-CTnI > 15.6 pg/ml, %	13.876 (2.888–66.673)	*0.001*
*Mode 2*		
Lymphocyte < 0.5 × 10^9^/L, %	6.496 (1.182–35.709)	*0.031*
D-dimer > 21 μg/mL, %	8.382 (0.770–91.279)	0.081
hs-CTnI > 15.6 pg/ml, %	14.140 (2.864–69.817)	*0.001*
White blood cell > 9.5 × 10^9^/L, %	2.883 (0.611–13.604)	0.181
*Mode 3*		
Lymphocyte < 0.5 × 10^9^/L, %	5.639 (1.027–30.958)	*0.047*
D-dimer > 21 μg/mL, %	6.556 (0.624–68.908)	0.117
hs-CTnI > 15.6 pg/ml, %	14.228 (2.841–71.252)	*0.001*
Neutrophil granulocyte > 6.3 × 10^9^/L, %	3.173 (0.712–14.129)	0.130
*Mode 4*		
Lymphocyte < 0.5 × 10^9^/L, %	6.560 (1.161–37.067)	*0.033*
D-dimer > 21 μg/mL, %	5.306 (0.490–57.501)	0.170
hs-CTnI > 15.6 pg/ml, %	8.860 (1.730–45.366)	*0.009*
PT > 14.5 s, %	4.719 (0.997–22.326)	0.050
*Mode 5*		
Lymphocyte < 0.5 × 10^9^/L, %	9.191 (1.190–70.996)	*0.033*
D-dimer > 21 μg/mL, %	24.142 (1.622–359.302)	*0.021*
hs-CTnI > 15.6 pg/ml, %	10.358 (1.711–63.036)	*0.011*
hs-CRP > 100 mg/L, %	19.528 (3.068–124.288)	*0.022*
*Mode 6*		
Lymphocyte < 0.5 × 10^9^/L, %	6.790 (1.242–37.129)	*0.027*
D-dimer > 21 μg/mL, %	7.964 (0.702–90.402)	0.094
hs-CTnI > 15.6 pg/ml, %	13.129 (2.668–64.599)	*0.002*
Albumin < 30 g/L, %	2.900 (0.660–12.746)	0.159
*Mode 7*		
Lymphocyte < 0.5 × 10^9^/L, %	7.598 (1.434–40.249)	*0.017*
D-dimer > 21 μg/mL, %	10.575 (0.752–148.749)	0.080
hs-CTnI > 15.6 pg/ml, %	13.942 (2.878–67.526)	*0.001*
Direct-bilirubin > 8umol/L	1.070 (0.121–9.442)	0.952
*Mode 8*		
Lymphocyte < 0.5 × 10^9^/L, %	8.684 (1.507–50.054)	*0.016*
D-dimer > 21 μg/mL, %	11.719 (1.086–126.460)	*0.043*
hs-CTnI > 15.6 pg/ml, %	13.209 (2.609–66.883)	*0.002*
Urea nitrogen > 8 mmol/L	4.233 (0.479–37.407)	0.194
*Mode 9*		
Lymphocyte < 0.5 × 10^9^/L, %	3.796 (0.671–21.481)	0.131
D-dimer > 21 μg/mL, %	16.923 (0.990–289.353)	0.051
hs-CTnI > 15.6 pg/ml, %	9.509 (1.833–49.323)	*0.007*
Procalcitonin > 0.05 ng/mL, %	9.876 (0.926–105.342)	0.058
*Mode 10*		
Lymphocyte < 0.5 × 10^9^/L, %	7.412 (1.205–45.602)	*0.031*
D-dimer > 21 μg/mL, %	4.625 (0.446–47.978)	0.199
hs-CTnI > 15.6 pg/ml, %	7.589 (1.360–42.335)	*0.021*
NT-proBNP ≥ 285 pg/ml, %	7.011 (1.452–33.843)	*0.015*

*OR* odds ratio, *PT* prothrombin time, *hs-CRP* high sensitivity C-reactive protein, *NT-proBNP* N-terminal pro-brain natriuretic peptide, *hs-CtnI* hypersensitive cardiac troponin I

**Fig. 1:**
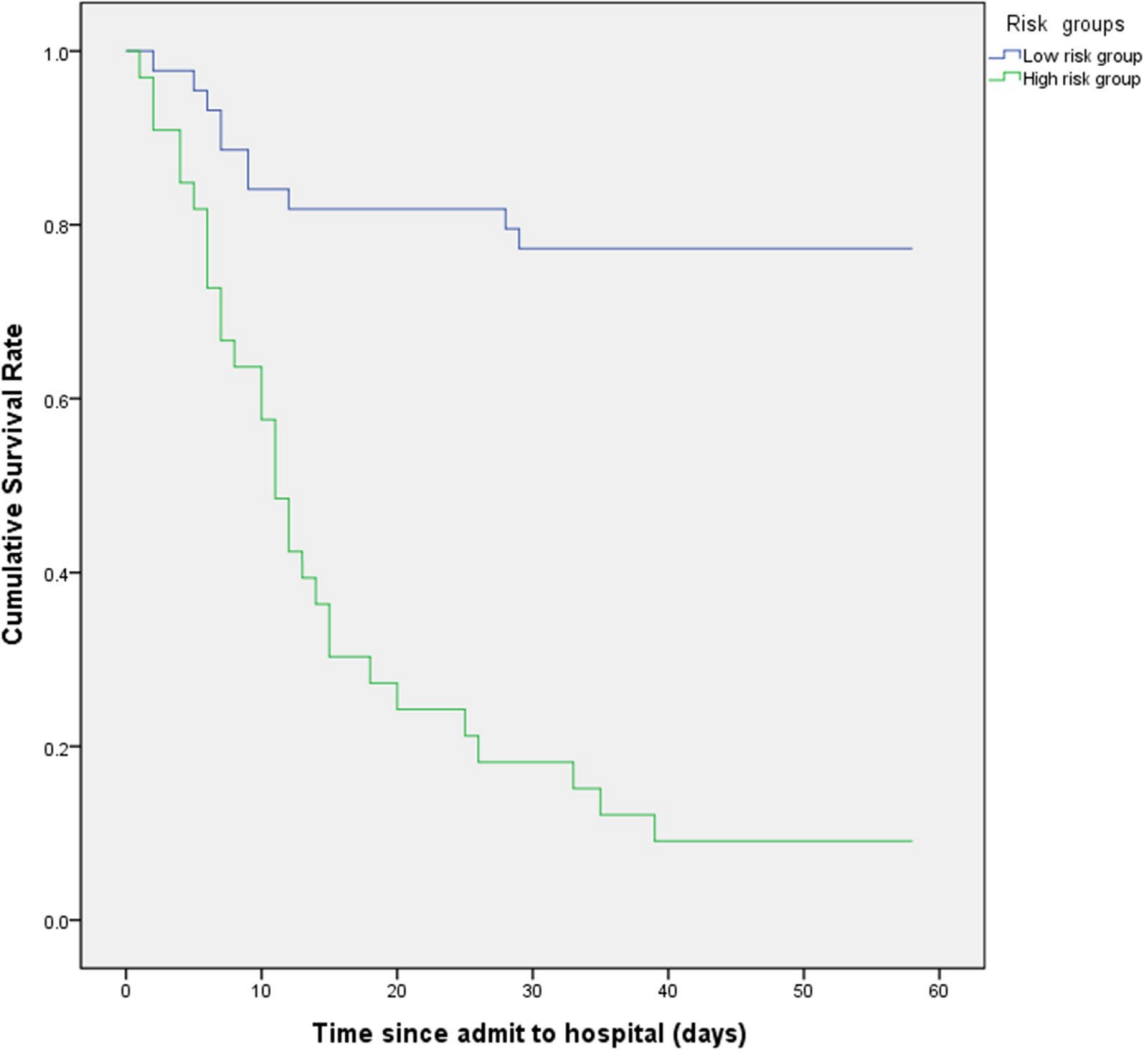
Survival of young adults with severe COVID-19. According to the level of lymphocytes, d-dimer, hs-CTnI and high sensitivity C-reactive protein (hs-CRP), we classified of young adults with severe COVID-19 into subgroups. Kaplan–Meier survival curves for survival rate during the time from admission to follow-up. The log-rank test was used to compare the Kaplan–Meier survival curves (Log-rank, P < 0.001)

According to the level of four variables in mode 5, we classified of young adults with severe COVID-19 to low-risk and high-risk subgroups. The cumulative survival rate of low-risk group was much higher than that of high-risk group (Fig. 1). The same predictive effect of four factors were shown in all-age participants with severe COVID-19.

## Discussion

This retrospective study reported clinical characteristics and identified several predictors for mortality of young adults with severe COVID-19. In particular, lymphocyte count less than 0.5 × 10^9^/L, d-dimer level greater than 21 μg/mL, hs-CTnI degree higher than 15.6 pg/ml and hs-CRP level higher than 100 mg/L were correlated with higher odds of on-admission mortality. Furthermore, we confirmed the markedly reduction of survival probability during the course of disease in severely ill young patients with high risk.

Formerly, elder age has been announced as an independent predictor of mortality in COVID-19 (Additional file 1: Table S1) [13]. More attention had been paid to older patients [14]. What’s more, clinical features of elderly patients with COVID-19 were significantly different from that of younger patients. Indeed, there are plenty of severe COVID-19 cases in young adults. However, clinical characters and risk factors of young adults with severe COVID-19 had not been fully understood.

Comparing with all-age patients, a lot of clinical factors such as sex, comorbidities and some clinical symptoms, showed no significant difference between non-survivors and survivors in severely young adults, indicating that characters of young adults were far more different from older patients in severe COVID-19 [12, 15]. It has to be noted that elderly patients were with more comorbidities, leading to more complicated pathogenesis in COVID-19 [8]. In severely young adults, comorbidities of non-survivors were similar to that of survivors. Alternatively, in all-age patients, the presence of unconscious and dizziness were higher in non-survivors than that of survivors, which were not observed in young adults of severe COVID-19. In the current study, dyspnea related to hypoxemia were more frequent in non-survivors, consistent with previous studies [16]. Correspondingly, non-survivors more often required mechanical ventilation and admission to Intensive Care Unit (ICU), indicating more prone to experience lung injury.

The pathogenesis of highly pathogenic human coronavirus is still not completely understood. Viral evasion of cellular immune responses and cytokine storm are thought to play important roles in disease severity [17]. Lymphopenia (< 0.5 × 10^9^/L) was found 45% in non-survivors, while 8% in survivors. The SARS-CoV-2 infection may impact on lymphocytes particularly, CD4 + T and CD8 + T cells, causing decrease in viral shedding [18, 19]. Increased levels of d-dimer (21 μg/mL), hs-CTnI (> 15.6 pg/mL) and hs-CRP (> 100 mg/L) were also correlated with a higher risk for death in multivariate logistic regression analysis. Increased levels of d-dimer, fibrin degradation products, and prolonged prothrombin time (PT) have been related with poor prognosis of COVID-19 patients [20]. Multiple pathogenetic mechanisms are included, such as Toll-like receptor activation, endothelial dysfunction, and tissue-factor pathway activation [21–23]. Myocardial insult is significantly associated with mortality of COVID-19, while the prognosis of patients with underlying coronary artery disease but without myocardial insult is relatively beneficial [24]. On the basis of recent studies, angiotensin-converting enzyme 2 (ACE2) is the human cell receptor with strong binding affinity to the Spike protein of SARS-CoV-2, and ACE2 is also highly expressed in heart [25, 26]. Thus, it is rational to hypothesize that COVID-19 induced cardiac injury might be mediated by ACE2. High level of hs-CRP, maker of systemic inflammation, was observed between severe and non-severe patients [15, 27]. As a classic acute phase protein, hs-CRP levels rise quickly responding to inflammation and might adjust the innate immune response by activating complement, stimulating the production of excessive inflammatory cytokines, or bonding fragment crystallizable region (Fc) receptors to activate phagocytosis [28, 29].

Among plenty of factors, lymphopenia, elevated level of d-dimer, hs-CTnT and hs-CRP were independently associated with COVID-19 mortality, suggesting multi-organ dysfunction in young adults of severe COVID-19. Further analysis showed that severely young adults with two or more factors abnormalities above would be more prone to death. Earlier identification of high-risk subgroup and subsequent timely appropriate therapy might improve outcomes. Though predictors of severely young adults showed great influence on mortality of COVID-19, the same effect of four predictors was comparable to that of all-age patients with severe COVID-19.

Tis study had some notable limitations too. Firstly, not all laboratory tests were done in all patients, due to the retrospective study design. Secondly, because the clinical observation of patients is still ongoing, some have not reached clinical end points. Thirdly, as an observational and retrospective study, we currently could not establish a validation cohort to evaluate the predictive effect due to the imperative timeline under this special situation.

## Conclusion

In conclusion, lymphopenia, elevated level of d-dimer, hs-CTnI and hs-CRP were independent predictors of mortality in young adults with severe COVID-19. Earlier confirmation, more intensive observation and appropriate treatment should be considered in high-risk young patients.

## Supplementary Information

**Additional file 1.** Additional Figure and Tables.

## Data Availability

All data generated or analysed during this study are included in this published article and its supplementary information files.

## Abbreviations

hs-CTnl: Hypersensitive cardiac troponin I
hs-CRP: High sensitivity C-reactive protein
SARS-CoV-2: Severe acute respiratory syndrome coronavirus 2
COVID-19: Coronavirus disease 2019
WHO: World Health Organization
IQR: Interquartile range
NT-proBNP: N-terminal pro-brain natriuretic peptide
OR: Odds ratio
ICU: Intensive Care Unit
ACE2: Angiotensin-converting enzyme 2
