# Supplementary material for: Clinical characteristics and predictors of mortality in young adults with severe COVID-19: a retrospective observational study
Source: Ann Clin Microbiol Antimicrob. 2021 Jan 6;20:3. doi: 10.1186/s12941-020-00412-9 (PMC7787410; doi:10.1186/s12941-020-00412-9)
Supplement: Supplementary file 1 — Additional file 1. Additional Figure and Tables. [file 12941_2020_412_MOESM1_ESM.doc]

**SUPPLEMENTARY INFORMATION**

**Clinical Characteristics and Predictors of Mortality for Young Adults with Severe COVID-19**

Yanjiao Lu^1^, Zhenli Huang^1^, Meijia Wang^1^, Kun Tang^1^, Shanshan Wang^1^, Pengfei Gao^2^, Jungang Xie^1^, Tao Wang^1^, Jianping Zhao^1^

^1^ Department of Respiratory and Critical Care Medicine, National Clinical Research Center of Respiratory Disease, Tongji Hospital, Tongji Medical College, Huazhong University of Science and Technology, Wuhan 430030, China.

^2^ Department of Respiratory and Critical Care Medicine, the First Affiliated Hospital of Henan University of Science and Technology, Luoyang, 471003, China. Tel: 86-27-83663596; Fax: 86-27-83662898.

Corresponding author full contact details:

Professor Jianping Zhao (E-mail: Zhaojp88@126.com) and Tao Wang (E-mail: wt7636@126.com), Department of Respiratory and Critical Care Medicine, National Clinical Research Center of Respiratory Disease, Tongji Hospital, Tongji Medical College, Huazhong University of Science and Technology, Wuhan 430030, China.

**Supplement figure 1***-* **Study flow diagram**

376 patients with confirmed COVID-19 as of Feb 15, 2020

197 patients with severe COVID-19

77 young patients with severe COVID-19

182 excluded

182 patients with nonsevere COVID-19

19 excluded

19 patients were still in hospital as of Mar 30, 2020

101 excluded

101 were older than 65 years old

178 patients with severe COVID-19

Supplement table 1. Characteristics of patients with severe COVID-19

|  | Total  n = 178 | Survivor  n = 71 | Non-survivor  n = 107 | P value |
| --- | --- | --- | --- | --- |
| Age, years | 67  (60-74) | 65  (57-72) | 68  (62-75) | **0.009** |
| Sex | .. | .. | .. | **0.013*** |
| Female | 68 (38%) | 35 (49%) | 33 (31%) | .. |
| Male | 110 (62%) | 36 (51%) | 74 (69%) | .. |
| Exposure history | 33 (18%) | 17 (25%) | 15 (14%) | 0.091* |
| Smoker | 14 (8%) | 5 (7%) | 9 (8%) | 0.700* |
| Comorbidity | 156 (88%) | 61 (86%) | 96 (89%) | 0.420* |
| Hypertension | 79 (45%) | 34 (49%) | 45 (42%) | 0.394* |
| Diabetes | 33 (19%) | 13 (18%) | 20 (19%) | 0.984* |
| Coronary heart disease | 23 (13%) | 8  (11%) | 15 (14%) | 0.616* |
| Chronic obstructive lung disease | 8 (5%) | 1 (1%) | 7 (7%) | 0.149* |
| Malignancy | 5 (3%) | 4 (6%) | 1 (1%) | 0.081* |
| Chronic kidney disease | 6 (3%) | 3 (4%) | 3 (3%) | 0.830* |
| Asthma | 1 (1%) | 1 (1%) | 0 (0) | 0.395* |
| Tuberculosis | 3 (2%) | 0 (0) | 3 (3%) | 0.279 |
| Bronchiectasis | 1 (1%) | 0 (0) | 1 (1%) | 1* |
| Chronic hepatitis B | 4 (2%) | 2 (3%) | 3 (3%) | 1* |
| Others | 65 (36%) | 27 (38%) | 39 (36%) | 1* |
| Respiratory rate, ＞30 breath per min | 43/176 (24%) | 9/70 (13%) | 34/106 (32%) | **0.003*** |
| Heart rate, ≥125 beats per min | 11/176 (6%) | 0/70  (0) | 11/106 (9%) | **0.004*** |
| Percutaneous oxygen saturation, ≤93% | 107 (60%) | 47 (66%) | 83 (76%) | 0.094* |
| Systolic pressure, mmH | 134  (121-149) | 132  (120-144) | 140  (124-151) | 0.502 |
| ＜90, mmHg | 4/177 (6%) | 0/71  (0) | 4/106 (4%) | 0.150* |
| Diastolic pressure, mmHg | 78.5  (67-85) | 76  (64-83) | 80  (70-86) | 0.089 |
| ＜60, mmH | 26/177 (15%) | 12/71 (17%) | 14/106 (13%) | 0.496* |

Data are median (IQR), n (%), or n/N (%). p values were calculated by Mann-Whitney U test, χ² test, or Fisher’s exact

test, as appropriate. *χ² test comparing all subcategories

Supplement table 2. Clinical symptoms of patients with severe COVID-19

|  | Total  n = 178 | Survivor  n = 71 | Non-survivor  n = 107 | P value |
| --- | --- | --- | --- | --- |
| Fever | 157 (89%) | 61 (86%) | 97 (91%) | 0.338 |
| Sore throat | 8 (5%) | 5 (7%) | 3 (3%) | 0.348 |
| Cough | 136 (78%) | 54 (76%) | 83 (78%) | 0.752 |
| Chest pain | 3 (2%) | 1 (1%) | 3 (3%) | 1 |
| Dyspnea | 113 (64%) | 36 (50%) | 78 (73%) | **0.003** |
| Fatigue | 92 (53%) | 38 (54%) | 54 (52%) | 0.785 |
| Myalgia | 27 (15%) | 13 (18%) | 15 (14%) | 0.369 |
| Nausea or vomiting | 6 (3%) | 1 (1%) | 5 (5%) | 0.436 |
| Diarrhea | 47 (26%) | 19 (26%) | 29 (27%) | 0.989 |
| Stomachache | 6 (3%) | 1 (1%) | 5 (3%) | 0.436 |
| Headache | 10 (6 %) | 3 (4%) | 7 (7%) | 0.723 |
| Unconscious | 12 (7%) | 1 (1%) | 11 (11%) | **0.042** |
| Dizziness | 8 (5%) | 0 (0) | 8 (8%) | **0.022** |

Data are n (%). p values were calculated by χ² test, or Fisher’s exact test, as appropriate. χ² test comparing all subcategories

Supplement table 3. Laboratory examinations at admission in patients with severe COVID-19

| Findings (normal range) | Total  n = 178 | Survivor  n = 71 | Non-survivor  n = 107 | P value |
| --- | --- | --- | --- | --- |
| Blood routine test |  |  |  |  |
| White blood cell, x10^9^/L (3.5-9.5) | 8.0  (5.7-11.4) | 6.5  (4.8-8.1) | 9.7  (6.9-13.4) | **<0.001** |
| Neutrophil granulocyte, x10^9^/L (1.8-6.3) | 7.1  (4.3-10.6) | 5.0  (3.7-6.6) | 9.1  (5.6-12.6) | **<0.001** |
| Lymphocyte, x10^9^/L (1.1-3.2) | 0.6  (0.5-0.9) | 0.8  (0.6-1.1) | 0.5  (0.4-0.7) | **<0.001** |
| Red blood cell, x10^9^/L (3.8-5.1) | 4.1  (3.8-4.6) | 4.1  (3.7-4.4) | 4.2  (3.8-4.7) | **0.013** |
| Haemoglobin, g/L (130-175) | 128  (117-141) | 125  (115-135) | 130  (119-145) | **0.031** |
| Platelet, x10^9^/L (125-350) | 171  (125-242) | 199  (153-276) | 159  (113-222) | **<0.001** |
| Coagulation function |  |  |  |  |
| PT, s (11.5-14.5) | 14.8  (14.0-16.2) | 14.3  (13.7-14.6) | 15.4  (14.4-17.1) | **<0.001** |
| APTT, s (29.0- 42.0) | 39.2  (36.1-44) | 40.5  (36.5-45.1) | 39.1  (35.6-43.6) | 0.383 |
| D-Dimer, ug/ml  (＜0.5) | 2.9  (1.2-21.0) | 1.8  (0.7-2.8) | 8.6  (1.7-21.0) | **<0.001** |
| Biochemical test |  |  |  |  |
| Albumin, g/L (35.0-52.0) | 30.9  (28.3-34.1) | 31.7  (29.9-35.0) | 30.1  (27.5-33.1) | **0.002** |
| Globulin, g/L (20.0-35.0) | 35.7  (31.8-40.0) | 34.4  (31.1-38.7) | 36.1  (33.2-39.2) | 0.059 |
| Aspartate aminotransferase, U/L (≤40) | 38 (27-56) | 30 (20-46) | 42 (30-64) | **0.001** |
| Alanine aminotransferase, U/L (≤41) | 29 (19-44) | 27 (16-45) | 29 (20-44) | 0.497 |
| Total-bilirubin, umol/L (≤26) | 11.7  (8.5-15.9) | 9.6  (7.5-13.3) | 12.8  (9.9-18.7) | **<0.001** |
| Direct-bilirubin, umol/L (≤8) | 5.4  (3.7-7.6) | 4.4  (3.4-6.5) | 6.2  (4.7-9.3) | **<0.001** |
| Creatinine, umol/L (59-104) | 82  (62.5-99.5) | 67  (56-85) | 89  (72-109) | **<0.001** |
| Urea nitrogen, mmol/L (3.1-8.0) | 6.6  (4.7-10.6) | 4.8  (3.1-6.3) | 8.4  (5.9-11.9) | **<0.001** |
| LDH U/L (135-225) | 478  (359-638) | 352  (272-460) | 564  (459-712) | **<0.001** |
| Infection-related biomarkers |  |  |  |  |
| Procalcitonin, ng/mL (0.02-0.05) | 0.2  (0.1-0.5) | 0.1  (0-0.2) | 0.3  (0.1-0.8) | **<0.001** |
| ERS, mm/h (0-15) | 38  (20.8-64.3) | 56  (27-72) | 36  (20-57) | **0.046** |
| Ferritin, ng (30-400) | 1245.8  (711-2047.8) | 738.7  (465.9-1334.2) | 1672.7  (974.3-2429.2) | **<0.001** |
| hs-CRP, mg/L (＜1) | 72.7  (38.5-140.2) | 47.2  (17.0-90.2) | 103.2  (53.4-169.1) | **<0.001** |
| Myocardial enzymes |  |  |  |  |
| Creatine kinase, U/L (≤190) | 134  (67-339) | 67  (39-153) | 177  (74-387) | **0.01** |
| NT-proBNP, pg/mL (<285) | 558  (188-1698) | 176  (74-478) | 843  (363-2568) | **<0.001** |
| hs-CTnl, pg/mL (≤15.6) | 19.6  (6.7-137.7) | 6.2  (2.8-15.2) | 41.9  (12.8-232.0) | **<0.001** |
| Myoglobin, ng/mL (≤106) | 113.3  (36.6-312.9) | 40.4  (26.4-135.4) | 198.2  (108-500.2) | **<0.001** |

Abbreviation: PT, prothrombin time; APTT, activated partial thromboplastin time; LDH, Lactate dehydrogenase; ERS, erythrocyte sedimentation rate; hs-CRP, high sensitivity C-reactive protein; NT-proBNP, N-terminal pro-brain natriuretic peptide; hs-CTnI, hypersensitive cardiac troponin I. Data are median (IQR), n (%), or n/N (%). p values were calculated by Mann-Whitney U test, χ² test, or Fisher’s exact test, as appropriate. *χ² test comparing all subcategories.

Supplement table 4.Treatment and outcomes in patients with severe COVID-19

|  | Total  n = 178 | Survivor  n = 71 | Non-survivor  n = 107 | P value |
| --- | --- | --- | --- | --- |
| Treatment |  |  |  |  |
| High-flow nasal cannula oxygen therapy | 39 (22%) | 9  (13%) | 30 (28%) | **0.015*** |
| Non-invasive mechanical ventilation | 85 (48%) | 6  (8%) | 79 (74%) | **<0.001*** |
| Invasive mechanical ventilation | 68 (38%) | 0  (6%) | 68 (64%) | **<0.001*** |
| ECMO | 2 (1%) | 0 (0) | 2 (2%) | 0.518* |
| Outcomes |  |  |  |  |
| ICU admission | 78 (43%) | 4  (6%) | 74 (69%) | **<0.001*** |
| ICU length of stay, days | 8  (4-12) | 28  (24-39) | 8  (4-12) | **<0.001** |
| Hospital length of stay, days | 18  (9-26) | 28  (22-39) | 10  (6-15) | **<0.001** |
| Time from illness onset to ICU admission, days | 16  (12-20) | 14  (7-17) | 16  (12-21) | 0.271 |
| Time from illness onset to death or discharge, days | 29  (20-40) | 41  (32-51) | 22  (17-28) | **<0.001** |
| SARS-CoV-2 RT-PCR test (-) | 91/177 (51%) | 69/71 (97%) | 22/107 (21%) | **<0.001*** |
| Duration of viral shedding after COVID-19 onset, days | 24  (18-29) | 25  (19-30) | 23  (17-24) | 0.144 |

Abbreviation: ECMO, extracorporeal membrane oxygenation; ICU, intensive care unit; RT-PCR, real-time polymerase chain reaction. Data are median (IQR), n (%), or n/N (%). P values were calculated by χ² test, or Fisher’s exact test, as appropriate. *χ² test comparing all subcategories.

Supplement table 5. Risk factors associated with mortality in severe COVID-19.

|  | Univariable OR  (95% CI) | P value | Multivariable  OR (95% CI) | P value |
| --- | --- | --- | --- | --- |
| Age, years | 1.038  (1.008-1.068) | **0.011** | 1.032  (0.989-1.077) | 0.145 |
| Sex(male) | 2.180  (1.172-4.054) | **0.014** | .. | .. |
| Respiratory rate ＞30 breath per min | 0.303  (0.135-0.681) | **0.004** | .. | .. |
| White blood cell ＞9.5x10^9^/L | 5.605  (2.707-11.603) | **<0.001** | .. | .. |
| Neutrophil granulocyte ＞6.3x10^9^/L | 7.557  (3.825-14.933) | **<0.001** | .. | .. |
| Lymphocyte ＜0.5x10^9^/L | 6.043  (2.727-13.390) | **<0.001** | 5.269  (1.881-14.759) | **0.002** |
| Platelet ＜100x10^9^/L | 3.034  (1.082-8.507) | **0.035** | .. | .. |
| PT ＞14.5s | 5.991  (3.098-11.583) | **<0.001** | .. | .. |
| D-dimer ＞21μg/mL | 9.521  (3.544-25.581) | **<0.001** | 4.492  (1.383-14.591) | **0.012** |
| Albumin ＜30g/L | 2.686  (1.405-5.134) | **0.003** | .. | .. |
| Direct-bilirubin ＞8umol/L | 4.077  (1.689-9.845) | **0.002** | .. | .. |
| Urea nitrogen ＞8mmol/L | 5.605  (2.707-11.603) | **<0.001** | .. | .. |
| Procalcitonin＞0.05ng/mL | 12.176  (4.370-33.920) | **<0.001** | .. | .. |
| hs-CRP ＞100mg/L | 3.745  (1.876-7.477) | **<0.001** | 3.777  (1.446-9.869) | **0.007** |
| Creatine kinase ＞190U/L | 3.636  (1.213-10.899) | **0.021** | .. | .. |
| NT-proBNP ≥285pg/ml | 7.504  (3.614-15.584) | **<0.001** | .. | .. |
| hs-CTnI ＞15.6pg/ml | 8.714  (4.107-18.489) | **<0.001** | 6.266  (2.652-15.383) | **<0.001** |

Abbreviation: OR, odds ratio; PT, prothrombin time; hs-CRP, high sensitivity C-reactive protein; NT-proBNP, N-terminal pro-brain natriuretic peptide; CTnI, hypersensitive cardiac troponin I.

Supplement table 6. AIC level of each multivariate logistic regression model.

| Model | AIC |
| --- | --- |
| 1 | -154.144 |
| 2 | -159.54 |
| 3 | -160.835 |
| 4 | -165.654 |
| 5 | -180.247 |
| 6 | -159.603 |
| 7 | -156.135 |
| 8 | -156.866 |
| 9 | -165.088 |
| 10 | -169.672 |
